# Oncofertility in the Age of HER2 Blockade, Immunotherapy, PARP inhibitors, CDK4/6 inhibitors and Endocrine Treatment: Unanswered Questions in Breast Cancer

**DOI:** 10.1007/s11864-026-01396-y

**Published:** 2026-05-29

**Authors:** Karolina Weiner-Gorzel, Lynda McSorley, Janice M. Walshe

**Affiliations:** 1 Department of Medical Oncology, St Vincent’s University Hospital, Dublin, Ireland; 2 Department of Medical Oncology, St Vincent’s Private Hospital, Dublin, Ireland; 3 School of Medicine, University College Dublin, Dublin, Ireland

**Keywords:** Oncofertility, Breast cancer, Fertility outcomes, Ovarian function, Pregnancy outcomes, Novel breast cancer agents, Targeted breast cancer treatment

## Abstract

As survival among reproductive-age patients with breast cancer continues to improve with modern therapies, concerns regarding fertility preservation, gonadotoxicity, and pregnancy safety have become increasingly prominent. Fertility considerations are well-recognized contributors to treatment refusal and premature discontinuation, therefore, oncofertility counselling should be initiated at diagnosis and integrated into therapeutic planning. Prompt referral for fertility preservation is essential for patients who may wish to conceive, without compromising oncologic outcomes. Systemic therapy selection should remain driven by tumour biology and recurrence risk; however, when clinically equivalent regimens are available, those with lower gonadotoxic potential are preferred. Cytotoxic chemotherapy is the main determinant of permanent ovarian insufficiency, and temporary ovarian suppression during chemotherapy should be routinely considered in appropriate candidates. Endocrine therapy is not associated with irreversible ovarian damage, but its long duration necessitates individualized planning for pregnancy, including supervised treatment interruption in selected low-risk patients. HER2-monoclonal antibodies should be delivered according to standard indications, as it does not appear to confer substantial additional ovarian toxicity beyond chemotherapy; however, pregnancy must be avoided during treatment. For antibody–drug conjugates, PARP inhibitors, and CDK 4 and 6 inhibitors, the absence of prospective human fertility data supports a precautionary approach, including pre-treatment fertility preservation, effective contraception during therapy, and adherence to recommended washout periods. Given the substantial gaps in clinical evidence, transparent communication of uncertainty is essential. The integration of standardized reproductive endpoints into clinical trials, alongside the development of predictive tools is critical to support evidence-based counselling and optimize long-term survivorship outcomes.

## Introduction

Breast cancer (BC) is the most common malignancy in women under 40 years of age, accounting for nearly one in five new cancer diagnoses in the United States [1]. As survival continues to improve with advances in systemic therapy [2, 3], attention has increasingly shifted toward survivorship issues—particularly fertility preservation, ovarian function, and pregnancy safety [4, 5]. This issue is becoming increasingly urgent. As childbearing is increasingly delayed worldwide, a growing proportion of women are diagnosed with BC before completing reproductive plans. Fertility concerns are therefore not only a survivorship issue but also a documented factor contributing to treatment refusal or premature discontinuation [6]. Accordingly, leading oncology societies and experts in the field—including the European Society for Medical Oncology (ESMO) and the American Society of Clinical Oncology (ASCO) recommend that fertility-related discussions be integrated into routine care for all reproductive-age patients [7–9].

While the gonadotoxic effects of cytotoxic chemotherapy are well established, the reproductive impact of modern targeted and immune-based therapies remains incompletely understood. Endocrine therapy, human epidermal growth factor receptor 2 (HER2)-targeted monoclonal antibodies and antibody–drug conjugates (ADCs), immune checkpoint inhibitors, poly(ADP-ribose) polymerase (PARP) inhibitors and cyclin-dependent kinase 4/6 (CDK4/6) inhibitors have transformed clinical outcomes across disease subtypes. However, pivotal trials rarely include fertility, amenorrhea, or menstrual recovery as prespecified endpoints, limiting the direct applicability of trial data to reproductive counselling.

In this context, ovarian function, fertility, pregnancy and neonatal outcomes are often discussed together, yet they represent related but biologically and clinically distinct domains. Ovarian function is commonly assessed using menstrual patterns and biomarkers of ovarian reserve such as anti-Müllerian hormone (AMH), whereas fertility refers to the ability to achieve a biological pregnancy. Neonatal outcomes reflect maternal and foetal safety following treatment exposure. Importantly, treatment-related amenorrhea or suppression of ovarian reserve markers does not necessarily indicate irreversible infertility or predict adverse pregnancy outcomes. Clear distinction among these endpoints is therefore essential when interpreting the reproductive effects of contemporary systemic BC therapies.

Emerging preclinical and translational data suggest that novel BC therapies may influence ovarian physiology through distinct mechanisms, including hormonal modulation, immune-mediated injury, DNA-repair interference, or cell-cycle disruption. Nevertheless, available clinical data remain limited, heterogeneous, and frequently derived from retrospective cohorts or small exploratory analyses, underscoring substantial uncertainty in this field.

**Fig. 1:**
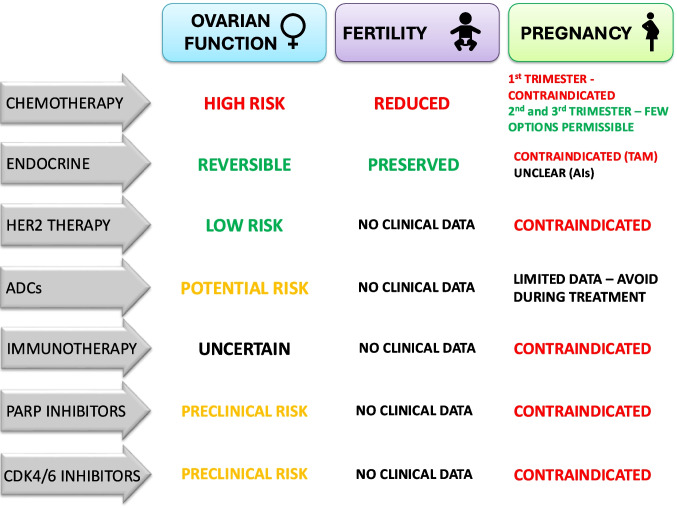
Impact of systemic breast cancer therapies on ovarian function, fertility, and pregnancy outcomes. Colour coding: red indicates high risk or contraindication; yellow/orange indicates potential or preclinical risk; green indicates low risk or reversible effects; black indicates insufficient clinical data. Further details and supporting evidence are provided in the main text of the review.

This review provides a comprehensive and up-to-date synthesis of the effects of systemic breast cancer therapies on ovarian function, fertility, and pregnancy-related outcomes in premenopausal women. Unlike prior reviews that typically focus on selected treatment modalities or fertility preservation strategies, the present work systematically evaluates all major classes of systemic therapies currently used in clinical practice, including endocrine therapies, HER2-targeted agents and antibody–drug conjugates, immune checkpoint inhibitors, PARP inhibitors, and CDK4/6 inhibitors. For each therapeutic class, both clinical and preclinical studies are examined in detail, with explicit discussion of biological mechanisms, reported reproductive outcomes, and existing evidence gaps. By integrating mechanistic insights with available clinical observations across the contemporary treatment landscape, this review aims to support informed oncofertility counselling in an era of rapidly expanding systemic treatment options. The mechanisms and clinical implications of these therapies on ovarian function, fertility, and pregnancy outcomes are summarized in Fig. 1.

## Methods

This article was conducted as a narrative, non-systematic review with the objective of synthesizing and contextualizing available clinical, translational, and preclinical evidence regarding the impact of contemporary systemic breast cancer therapies on ovarian function, fertility outcomes, and pregnancy safety in premenopausal women.

A literature search was performed using PubMed, Embase, as well as official websites hosting conference proceedings from the American Society of Clinical Oncology, the European Society of Medical Oncology and the American Association for Cancer Research**.** Publications from database inception through December 2025 were considered**.** Search terms included combinations of keywords and Medical Subject Headings (MeSH) such as *breast cancer*, *oncofertility*, *ovarian function*, *amenorrhea*, *anti-Müllerian hormone*, *fertility outcomes*, *gonadotoxicity*, *pregnancy*, and *neonatal outcomes*, alongside drug-class–specific terms including *endocrine therapy*, *tamoxifen*, *aromatase inhibitors* (*letrozole, anastrozole)*, *HER2-targeted therapy*, *trastuzumab*, pertuzumab, *antibody–drug conjugates*, *immune checkpoint inhibitors*, *pembrolizumab*, *PARP inhibitors* (*olaparib, talazoparib) and CDK4/6 inhibitors (ribociclib, palbociclib, abemaciclib)*.

Reference lists of relevant articles and prior reviews were manually screened to identify additional pertinent studies. Conference abstracts were cross-referenced to identify corresponding full-text publications. Additional relevant studies cited within included articles but not retrieved through the initial search strategy were also considered. Case reports and case series were included when higher-level evidence was unavailable, particularly for pregnancy exposures.

Inclusion criteria were intentionally broad to reflect the heterogeneity of available evidence and included studies reporting on ovarian function (e.g., amenorrhea, hormonal markers), fertility outcomes, or pregnancy and neonatal safety in the context of systemic breast cancer therapies. Non-English-language publications, conference abstracts without sufficient methodological detail, and studies unrelated to breast cancer were excluded, with few exceptions where breast cancer-specific studies on related topic are not available.

As a narrative review, no formal quality scoring or meta-analytic synthesis was performed. Instead, evidence was critically appraised and stratified by drug class and level of evidence**,** with explicit distinction between clinical, translational, and preclinical findings. The intent was to summarize established knowledge, identify areas of uncertainty, and delineate key gaps relevant to clinical counselling and future research in oncofertility.

## Endocrine Therapy

Endocrine therapy is the cornerstone of treatment for hormone receptor (HR)–positive BC. Standard treatment includes tamoxifen and aromatase inhibitors (AIs)—such as anastrozole, letrozole, and exemestane—with or without ovarian function suppression (OFS), depending on menopausal status [10]. These agents substantially reduce recurrence and improve survival. A meta-analysis of over 20,000 patients demonstrated that five years of tamoxifen reduces the risk of recurrence by 47% and BC mortality by approximately one third over 15 years [11]. Likewise, AIs further reduce recurrence rates by about 30% compared with tamoxifen in postmenopausal women [12].

### Tamoxifen—Preclinical and Mechanistic Data

Tamoxifen acts as an anti-oestrogen in breast tissue but exhibits partial agonist activity in the ovaries and endometrium, occasionally leading to ovarian hyperstimulation [13]. Although the mechanism is not completely understood, it likely involves tamoxifen-driven disruption of the negative pituitary feedback loop, thereby activating follicle-stimulating hormone (FSH)-driven ovarian steroidogenesis [14]. Direct effects on granulosa cells, including modulation of oestrogen production and luteinizing hormone (LH) receptor expression, have also been described [15, 16]. Reported hormonal changes during tamoxifen therapy are inconsistent [14, 17–21], which limits the interpretability of hormonal markers during active tamoxifen therapy.

### Tamoxifen—Clinical Data

Tamoxifen is frequently associated with amenorrhea. In a retrospective cohort of 250 premenopausal women with ductal carcinoma in situ (DCIS), amenorrhea occurred in 48% of tamoxifen—only treated patients compared with 15% of controls, with older patient age as predictor [22]. Importantly, it has not been linked to an earlier menopause, and menstrual effects are generally reversible [14].

The impact of tamoxifen on treatment-related amenorrhea (TRA) after chemotherapy has been extensively investigated (Table 1). Studies consistently report higher TRA rates with tamoxifen after chemotherapy (Table 1) [23–26]. Meta-analyses confirm the association, demonstrating a significantly increased risk of TRA when tamoxifen is administered following chemotherapy [27, 28].

**Table 1:** Tamoxifen-induced amenorrhea

Study	Design	Number of participants	Treatment	Findings	*p*
International Breast Cancer Study Group (Trial 13–93) [23]	Prospective	1246	Chemotherapy (AC/EC) ± Tam. Tam group *n* = 365, no Tam group *n* = 370	At 15 months post chemotherapy Tam increased TRA compared to no TAM (88% vs 84%)	-
Poorvu (The Young Women’s Breast Cancer Study) [29]	Prospective cohort	789	Tam use following chemotherapy (various regimens). Tam group *n* = 459, no Tam group *n* = 296	TRA occurred in 39.7% of participants treated with Tam vs 30.1% not receiving Tam at 1 year (OR 1.55 95%CI 1.12–2.16	0.009
Swain (NSABP-30) [24]	Prospective trial	708	Chemotherapy ACT (Doxorubicin/cyclophosphamide/docetaxel) ± Tam	TAM increased the rate of TRA post chemotherapy at 1 year (69% vs 60%)	0.003
Goodwin [25]	Prospective study	183	Chemotherapy (45%), chemotherapy + Tam (13%), Tam only group (12%). Tam group *n* = 47	Tam increased TRA when given with chemotherapy (84% vs 64.6%)	0.001
Petrek [30]	Prospective study	426	Chemotherapy (87%) with addition of Tam (55%). Tam group *n* = 329	Tam increased amenorrhea by 15% at 1 and 2 years regardless of chemotherapy regimen	-
Abusef [31]	Prospective cohort	431	Tam use following chemotherapy (AC/AC-T ± trastuzumab). Tam group *n* = 303	Women treated with Tam post-chemotherapy were twice as likely to remain amenorrheic (OR, 2.12; 95% CI, 1.13–4.00) at 1 year	-
Zhou [26]	Prospective study	170	Tam following chemotherapy (FEC/TE/NE)	Tam increased TRA in univariate (44.44% vs 16.98%) and multivariate analysis OR 3.69	0.001 0.003
Pourali [32]	Prospective cohort	119	Tam following chemotherapy (12 month follow up)	Tam significantly correlated with TRA (45% vs 25%)	0.0001
Kim (Astrra trial) [33]	Prospective	1067	Tam following chemotherapy	28% of patients recovered menstruation 2–5 years post chemotherapy while on Tam	
Chien [22]	Retrospective cohort	250	Tam (*n* = 125) vs control (*n* = 125) in DCIS	Tam induced amenorrhea comparing to control group (48% vs 15%)	< 0.001
Berliere [14]	Retrospective	138	Tam (*n* = 68) and Tam + chemotherapy (*n* = 70)	In the Tam only group, menstrual pattern is associated with hormonal levels	-
Park [34]	Retrospective	872	Tam following chemotherapy	Lack of Tam predicted continued menses (HR 0.4, 95% CI 0.3–0.5)	< 0.001
Wang [27]	Meta-analysis	-	Tam following chemotherapy	Tam significantly increased TRA risk (OR 0.568, 95% CI 0.461–0.71)	< 0.001
Zavos [28]	Meta-analysis	2953	Tam following chemotherapy	Two fold increased risk of TRA (OR 1.9, 95% CI 1.45–2.48)	

Abbreviations: *Tam* tamoxifen; *AC* Doxorubicin/Cyclophosphamide; *EC* Epirubicin/Cyclophosphamide; *AC-T* Doxorubicin/Cyclophosphamide/Paclitaxel; *FEC* Fluorouracil/Epirubicin/Cyclophosphamide; *TE* Docetaxel/Epirubicin. Sample sizes are reported as described in the original publications. Where available, group-level sample sizes are indicated. Many included studies were retrospective or exploratory in nature and did not report fertility-specific comparator groups or clearly specify group-level patient numbers

### Tamoxifen – Ovarian Function and Pregnancy

Importantly, ovarian function recovers following tamoxifen cessation or after a sufficient washout interval. In a population-based cohort of 397 BC survivors, while prior tamoxifen use was associated with lower parity these women had higher AMH levels and antral follicle counts compared with non-users [35]. These observations suggest no direct detrimental effect on ovarian reserve; rather, post-treatment fertility outcomes may reflect behavioural and psychosocial factors. Notably, tamoxifen has also been used successfully in controlled ovarian stimulation protocols for oocyte retrieval before chemotherapy, yielding favourable reproductive outcomes [36]. Overall, tamoxifen appears to transiently alter ovarian function, with recovery after discontinuation.

Pregnancy safety data are limited and derived primarily from animal studies, case reports, and retrospective series. Available evidence suggests a higher incidence of congenital anomalies in tamoxifen-exposed pregnancies compared with rates in the general population (12.6% vs 3.9%), particularly skeletal and craniofacial malformations [37, 38].

Given the risks, tamoxifen is contraindicated during pregnancy, and a two- to three-month washout period before conception is widely recommended. Effective contraception and comprehensive preconception counselling are essential, while long-term offspring safety data remain limited.

### Aromatase Inhibitors – Mechanistic and Clinical Data

Third-generation AIs, comprising non-steroidal agents (anastrozole and letrozole) and the steroidal agent exemestane, inhibit aromatase—the rate-limiting enzyme in oestrogen biosynthesis. In postmenopausal women, aromatase activity in the adrenal glands, skin, muscle, and adipose tissue is the principal source of circulating oestrogens [39].

AIs profoundly suppress circulating oestrogen levels and alter gonadotropin profiles. Prospective and observational studies consistently demonstrate marked reductions in oestradiol and estrone, accompanied by compensatory increases in FSH and androgens [40–43]. These effects may vary according to patient factors such as BMI. When used for fertility preservation in BC patients after surgery or chemotherapy, letrozole increases FSH levels significantly more than tamoxifen [44]. During in vitro stimulation, both anastrozole and letrozole almost completely suppress oestradiol production [45].

Indirect evidence of preserved ovarian function comes from studies demonstrating recovery of ovarian activity during AI therapy in women with chemotherapy-induced amenorrhea, with recovery rates ranging from 12 to 39% [46–48]. Recovery rates were higher in patients younger than 40 years old.

In fertility preservation settings, both letrozole and anastrozole are widely incorporated into ovarian stimulation protocols for oocyte or embryo cryopreservation in BC patients, demonstrating proven safety and efficacy [45, 49–51]. Preclinical rodent studies support these findings, showing that letrozole enhances FSH receptor expression, promotes follicle growth, and may induce a polycystic ovary–like phenotype, consistent with its ability to stimulate folliculogenesis in fertility protocols [52, 53].

### Aromatase Inhibitors—Pregnancy

Clinical evidence also supports the reproductive safety of letrozole exposure in pregnancy. Large cohort studies assessing neonatal outcomes following assisted reproduction have demonstrated no increase in congenital malformations compared with the general population [54–57].

#### Clinical Take-Home Message

Tamoxifen transiently alters hypothalamic–pituitary signalling and ovarian reserve markers, with reversible effects on menstrual function and a potential teratogenic risk during pregnancy. In contrast, AIs do not appear directly gonadotoxic and are routinely employed in fertility preservation protocols, with reassuring evidence supporting neonatal safety following conception with letrozole exposure (Table 2).

**Table 2:** Summary of endocrine therapy mechanism, effects, ovarian function, fertility outcomes and pregnancy safety

	Tamoxifen	Aromatase Inhibitors
Mechanism of action	Oestrogen antagonist in breast tissue and partial agonist in ovaries and endometrium. Disrupts pituitary feedback, increasing FSH-driven steroidogenesis and oestradiol levels	Inhibit aromatase, the rate-limiting enzyme in oestrogen biosynthesis, blocking peripheral conversion of androgens to oestrogens
Endocrine effect	Disrupts pituitary feedback, increasing FSH-driven steroidogenesis and oestradiol levels. Variable hormonal effects are reported between studies with high patient to patient variability	Reported to consistently and profoundly and suppress oestrogen and increase gonadotropin levels
Ovarian function effect	Frequently leads to amenorrhea in up to 40% of patients and increases rates of TRA following chemotherapy treatment	No evidence of direct gonadotoxicity. In patients with chemotherapy-induced amenorrhea, allows for menstrual or biochemical ovarian function recovery during therapy in 12–39% of patients
Fertility and ovarian reserve	No consistent evidence of permanent ovarian damage. Ovarian function typically recovers post-treatment or after washout. Used successfully in ovarian stimulation for fertility preservation	Widely used in fertility preservation protocols. Safe for ovarian stimulation. No evidence of reduced oocyte yield or ovarian reserve impairment
Pregnancy and neonatal outcomes	Teratogenic potential observed in animal and human data. Reported congenital anomalies (12.6% vs 3.9% in general population), particularly skeletal and craniofacial defects. Washout period of 2–3 months before conception is recommended	Reproductive safety well established. Large cohorts (> 4,000 pregnancies) show no increase in congenital malformations. Neonatal outcomes comparable to general population (2.1% congenital anomaly rate)

This table provides a narrative summary of key reproductive findings across studies. Detailed study characteristics, methodologies, and original data sources are cited and discussed in the corresponding sections of the main text

## Her2-Targeted Monoclonal Antibodies

HER2–positive BC accounts for approximately 15–20% of all BC and is characterized by aggressive biology [58]. The introduction of HER2-targeted monoclonal antibodies such as trastuzumab and pertuzumab has markedly improved survival in both early-stage and metastatic disease [58].

### HER2-Targeted Monoclonal Antibodies Mechanism

Trastuzumab and pertuzumab are humanized monoclonal antibodies directed against the HER2 receptor. Trastuzumab binds to the extracellular domain IV of HER2, inhibiting downstream signalling, whereas pertuzumab binds extracellular domain II, preventing dimerization with other HER family receptors, particularly HER3, thereby further suppressing tumour-promoting pathways [59].

### HER2-Targeted Monoclonal Antibodies and Ovarian Function and Fertility, Clinical Data

The effect of HER2-targeted monoclonal antibodies on ovarian function remains incompletely defined; however, available data consistently suggest no additional gonadotoxicity beyond chemotherapy (Table 3). Across randomized and observational studies—including NRG Oncology/NSABP B-47 and multiple retrospective cohorts—trastuzumab was not associated with increased rates of treatment-related amenorrhea (TRA) compared with chemotherapy alone [31, 60, 61]. Small studies evaluating ovarian reserve markers likewise demonstrated no significant impact of trastuzumab on AMH levels, although interpretation is limited by heterogeneity and sample size [61].

**Table 3:** HER-2-targeted monoclonal antibodies and amenorrhea

Study	Design	Number of participants	Treatment	Findings	*p*
NRG Oncology/NSABP B-47 [60]	Phase III randomized	1428	Chemotherapy ± trastuzumab	No significant difference in TRA between trastuzumab and control arms (84% vs 86.3%) at 12 months post-treatment	0.2
Abusef [31]	Retrospective cohort	431	AC only (61%) vs AC-T (39%), 14% received trastuzumab	No difference in TRA rates at 12 months post-treatment (OR 0.6 95% CI 0.22–1.61)	-
Morarji [61]	Retrospective	100	Chemotherapy ± trastuzumab (various regimens)	No effect of trastuzumab on serum AMH levels observed small heterogenous sample limits interpretation	0.03
Lambertini (ALLTO trial) [62]	Randomized phase III	2862	Four adjuvant arms: trastuzumab, lapatinib, sequential trastuzumab- > lapatinib, trastuzumab + lapatinib (all with chemotherapy)	No significant differences in TRA across arms (72.6%, 74%, 72.1% and 74.8%) at 37 months from therapy initiation	0.64
Ruddy (APT trial) [63]	Retrospective	64	Weekly paclitaxel + trastuzumab (12 weeks), followed by 9 months of trastuzumab	Amenorrhea observed in 13% at 18 months and 28% at 51 months post-treatment (95% CI 18–41%)	-
Lambertini (NeoALTTO trial) [64]	Prospective translational	130	Two-week trastuzumab or lapatinib monotherapy before chemotherapy (biological window study)	Small but significant decline in AMH after 2 weeks of trastuzumab/lapatinib AMH nearly undetectable after paclitaxel addition	< 0.01
Ruddy (ATEMPT trial) [66]	Randomised phase II	123	T-DM1 vs TH (paclitaxel + trastuzumab)	TRA rates lower with T-DM1 compared to TH (24% vs 50% at 18 months, suggests lower gonadotoxicity with T-DM1	0.045
Silva [19]	Retrospective	-	Chemotherapy ± trastuzumab	Trastuzumab associated with improved AMH recovery and higher rate of menstrual resumption vs chemotherapy alone	0.046
Levi [65]	Preclinical (mouse model) + translational		Trastuzumab ± chemotherapy	Trastuzumab showed potential ovarian protection via vascular stabilisation and reduced apoptotic injury	-

Sample sizes are reported as described in the original publications. Where available, group-level sample sizes are indicated. Many included studies were single arm, retrospective or exploratory in nature and did not report fertility-specific comparator groups or clearly specify group-level patient numbers

Additional studies have evaluated TRA in HER2-targeted therapy arms, although without direct non-HER2 comparator groups [62–64]. In the ALLTO trial, TRA rates between four adjuvant arms: trastuzumab, lapatinib, sequential trastuzumab → lapatinib, and trastuzumab + lapatinib, were similar [62]. However, the design precluded comparison with non-HER2-targeted regimens. Additional data from single-arm and translational studies support these findings. In the APT trial, amenorrhea rates remained relatively low following paclitaxel plus trastuzumab [63].

In contrast, the NeoALTTO “biological window” study demonstrated a modest short-term decline in AMH after brief exposure to trastuzumab or lapatinib, followed by a marked reduction after chemotherapy, indicating that cytotoxic agents remain the primary driver of ovarian toxicity [64]. Although the two-week exposure period was insufficient to define long-term ovarian function, the findings suggest an early biological effect of HER2-targeted monoclonal antibodies on ovarian activity.

Some data suggest trastuzumab may facilitate ovarian recovery when combined with chemotherapy. Small clinical studies have demonstrated improved AMH recovery and higher rates of menstrual resumption in patients receiving trastuzumab [19, 65]. Preclinical data by Levi et al. further support a protective effect of trastuzumab on ovarian reserve, mediated by vascular stabilization and reduced apoptotic injury [65].

To date, no clinical studies have specifically examined the effect of pertuzumab alone on fertility. Its impact remains difficult to isolate, as pertuzumab is almost exclusively administered alongside trastuzumab, which continues to serve as the backbone of HER2-targeted monoclonal antibodies.

### HER2-Targeted Monoclonal Antibodies and Pregnancy, Clinical Data

Pregnancies, including successful pregnancies, have been documented during HER2-directed therapy, although reports remain sporadic [67–69]. Pregnancies have been reported during or shortly after trastuzumab exposure in clinical trials (e.g., HERA, ALTTO), outcomes remain heterogeneous, with increased rates of spontaneous abortion but no consistent signal for congenital malformations in limited datasets [70, 71].

However, pregnancy during active anti-HER2 monoclonal antibody therapy is associated with significant maternal and foetal risk. A large case–control study demonstrated substantial toxicity associated with trastuzumab and pertuzumab, including oligohydramnios, congenital respiratory anomalies, neonatal renal failure, cardiac malformations, and intrauterine growth restriction, especially when exposure occurred during the second or third trimester [72, 73]. Consequently, continuation of anti-HER2 monoclonal antibody therapy during pregnancy is contraindicated, and treatment should be discontinued upon pregnancy diagnosis. By contrast, conception after completion of anti-HER2 monoclonal antibody therapy appears safe and is not associated with adverse outcomes. In the NeoALTTO and ALTTO trials, pregnancies occurring after therapy cessation resulted in healthy live births, except for one case of trisomy 21 in a 38-year-old woman previously exposed to trastuzumab and lapatinib more than three years prior to conception [71].

#### Clinical Take-Home Message

Current evidence suggests that trastuzumab is less gonadotoxic than chemotherapy and may even promote ovarian recovery. Pregnancy during active anti-HER2 monoclonal antibody treatment carries significant risk and should be avoided, whereas conception after treatment completion appears safe and is not associated with adverse maternal or foetal outcomes (Table 4).

**Table 4:** Summary of HER2-targeted monoclonal antibodies on ovarian function, fertility and pregnancy

	HER2-tergeted monoclonal antibodies (trastuzumab, pertuzumab)
Mechanism of action	Monoclonal antibodies targeting HER2 receptor. Trastuzumab binds domain IV, blocking downstream signalling; pertuzumab binds domain II, preventing HER2/HER3 dimerization
Endocrine effect	No direct endocrine disruption reported. HER2 blockade does not affect hypothalamic–pituitary signalling or gonadotropin levels
Ovarian function effect	No increase in treatment-related amenorrhea compared with chemotherapy alone (TRA 84–86%). Some evidence suggests trastuzumab may support ovarian recovery post-chemotherapy
Fertility and ovarian reserve	Limited data; no consistent evidence of direct gonadotoxicity. Small studies indicate potential AMH recovery and menses resumption after trastuzumab. No human data on pertuzumab monotherapy
Pregnancy and neonatal outcomes	Pregnancy during active HER2-targeted monoclonal antibody therapy linked to maternal and foetal toxicity (oligohydramnios, renal failure, cardiac malformations). Post-treatment conceptions appear safe, with live births and no increase in congenital anomalies reported. Anti-HER2 monoclonal antibody therapy is contraindicated during pregnancy; conception recommended ≥ 7 months after last dose

This table provides a narrative summary of key reproductive findings across studies. Detailed study characteristics, methodologies, and original data sources are cited and discussed in the corresponding sections of the main text

### Antibody–Drug Conjugates

Antibody–drug conjugates (ADCs) represent an emerging and increasingly relevant therapeutic class in BC, combining the specificity of monoclonal antibodies with the cytotoxic activity of chemotherapy payloads. Agents such as trastuzumab emtansine (T-DM1), trastuzumab deruxtecan (T-DXd), and sacituzumab govitecan deliver potent cytotoxic agents directly to tumour cells following receptor-mediated internalization. T-DM1 consists of trastuzumab covalently linked via a stable linker to the microtubule inhibitor emtansine, with an average drug-to-antibody ratio (DAR) of 3.5. T-DXd is an ADC composed of trastuzumab conjugated to deruxtecan, a topoisomerase I inhibitor, with a higher DAR of approximately 8 [74]. Sacituzumab govitecan is a trophoblast cell-surface antigen-2 (Trop-2)–directed antibody–drug conjugate delivering SN-38, a topoisomerase I inhibitor, which induces DNA damage and tumour cell death following internalization and intracellular payload release [75, 76].

From an oncofertility perspective, ADCs raise unique concerns due to their dual nature. While the antibody component confers tumour selectivity, the cytotoxic payload retains the potential for off-target effects, including gonadotoxicity. Preclinical and mechanistic data indicate that ADC payloads may induce off-target cellular injury through systemic release or bystander effects [77]. By analogy with conventional chemotherapy, these mechanisms raise concern for potential impairment of ovarian reserve through follicular depletion and DNA damage. To date, limited clinical insight is available. The ATEMPT trial evaluated menstrual function in 123 premenopausal women randomized to T-DM1 or paclitaxel plus trastuzumab (TH). Rates of chemotherapy-related amenorrhea were significantly higher in the TH arm compared with T-DM1 (50% vs 24%) at 18 months [66]. Interestingly, the TRA rate following TH was higher than in prior reports [29, 75]. These findings suggest a potentially more favourable gonadotoxicity profile of T-DM1 compared with standard chemotherapy plus trastuzumab, although interpretation is limited by the absence of direct ovarian reserve markers.

In contrast, although T-DXd is increasingly used in younger patients with breast cancer, no clinical data are available regarding its effects on ovarian function, fertility, or pregnancy outcomes. While HER2-targeted monoclonal antibodies are associated with adverse pregnancy outcomes, as discussed above, the topoisomerase I inhibitor payload (deruxtecan) is a DNA-damaging agent with potential gonadotoxic effects based on preclinical and chemotherapy data [78]. Given its distinct payload and linker characteristics, extrapolation of reproductive safety data from T-DM1 to T-DXd is not appropriate, and dedicated clinical data are needed.

Similarly, for sacituzumab govitecan, no clinical data on ovarian function, fertility, or pregnancy outcomes have been reported in pivotal trials (including ASCENT and TROPiCS-02). However, animal studies indicate potential reproductive toxicity, including increased follicular atresia, uterine changes, and genotoxic effects related to the SN-38 payload [79].

With the rapid development and earlier integration of ADCs into clinical practice, including in the adjuvant setting, systematic evaluation of their reproductive safety is urgently needed. Factors such as payload class, drug–antibody ratio, bystander effect, and drug release kinetics may influence gonadal exposure, underscoring the need to incorporate fertility endpoints into clinical trials.

At present, in the absence of robust clinical evidence, ADCs should be considered as potentially gonadotoxic, and fertility preservation strategies should be discussed prior to treatment initiation. Further translational and prospective clinical studies are required to better define their reproductive safety profile.

#### Clinical Take-Home Message

ADCs likely carry a risk of gonadotoxicity due to their cytotoxic payloads, but evidence remains limited; therefore, a precautionary approach with early fertility counselling is warranted.

## Immunotherapy

Immunotherapy (IO), particularly pembrolizumab, has become a standard of care for early-stage and metastatic triple-negative breast cancer (TNBC) [80]. Pembrolizumab is a monoclonal antibody targeting programmed death-1 (PD-1), which enhances antitumour immunity by restoring T-cell function and promoting tumour-specific cytotoxicity [81].

Despite its expanding use across solid tumours and increasing FDA-approved indications, little is known about its impact on fertility. Clinical trials rarely report gonadotoxicity or reproductive outcomes, and oncofertility counselling before IO initiation remains suboptimal, estimated at 40% [82]. Two key areas of concern have emerged: (1) direct ovarian effects through immune-mediated injury or local immunomodulation, and (2) indirect hormonal effects via immune-related endocrinopathies [83].

### Immunotherapy and Gonadotoxicity – Preclinical Data

Preclinical data, though limited, suggest potential reproductive toxicity [84–86]. Walter et al. showed that ipilimumab, an anti-cytotoxic T-lymphocyte associated protein 4 (anti–CTLA-4) binds ovarian connective tissue in primates without altering oocyte morphology [84]. In murine models, blockade of PD-1/PD-L1 and CTLA-4 pathways significantly reduced primordial follicle numbers through immune-mediated mechanisms [85, 86].

### Immunotherapy and Gonadotoxicity – Clinical Data

Clinically, only one study has investigated ovarian toxicity in BC. Specifically, in 79 premenopausal TNBC patients, the addition of pembrolizumab to chemotherapy (carboplatin/paclitaxel/epirubicin/cyclophosphamide) did not significantly alter AMH, FSH, or oestradiol levels one year post-treatment compared with chemotherapy alone [87]. In melanoma, ipilimumab treatment in 28 stage III patients reduced AMH, oestradiol, and LH but did not affect FSH or prolactin [88]. Similarly, 14 stage III/IV melanoma patients treated with IO demonstrated decreased AMH and antral follicle counts post-therapy [89]. Pharmacovigilance analyses reported an increased risk of genital tract fistulae with atezolizumab and pembrolizumab, but not nivolumab [90].

A major concern relates to IO-induced endocrine dysfunction affecting the hypothalamic–pituitary–gonadal axis. Immune-related endocrinopathies can disrupt thyroid-stimulating hormone (TSH), adrenocorticotropic hormone (ACTH), FSH, and LH secretion [91]. In melanoma, up to 11% of ipilimumab-treated patients developed hypophysitis, with over 80% experiencing gonadotropin deficiency [92, 93]. In TNBC, KEYNOTE-522 clinical trial reported immune-related adverse events (irAEs) in 35% of pembrolizumab-treated patients versus 13% with chemotherapy, with hypophysitis observed in 1.9% [94]. Real-world data have confirmed irAEs in 31% of patients with early TNBC receiving IO, most commonly thyroiditis (9.2%), adrenal insufficiency (1.9%), and hypophysitis (1.4%) [95]. These endocrine toxicities are well-recognized causes of infertility and maternal–foetal complications [1].

Unlike chemotherapy, IO-related toxicity may appear long after treatment completion [96, 97]. A recent analysis reported a median onset of 167 days after IO cessation, with events occurring up to 294 days later [96]. Similarly, a cohort of nearly 800 IO-treated patients found that 10.8% required hospitalization for irAEs more than one year post-exposure [97]. For pembrolizumab, current NCCN, ASCO, and ESMO guidelines recommend avoiding pregnancy during IO and for at least four months following completion of treatment [98, 99]. Recent data may suggest even more precaution in delaying conception for at least one year following immune checkpoint inhibitor treatment, notably, a strategy driven by uncertainty regarding delayed immune-related adverse events rather than by evidence-based thresholds.

### Immunotherapy and Pregnancy

Pregnancy poses additional risks due to the critical role of CTLA-4/CD80/CD86 and PD-1/PD-L1 pathways in maintaining maternal–foetal tolerance [100]. In murine models, PD-L1 blockade significantly increased miscarriage rates [101]. Human evidence remains sparse. Pharmacovigilance data from WHO’s VigiBase® identified 103 reports of IO exposure during the peri-pregnancy period with no statistically significant increase in adverse outcomes, though confounding and reporting bias limit interpretation [102]. Conversely, a systematic review found higher rates of pregnancy complications, low birth weight, and prematurity following IO exposure [73]. Case reports describe severe immune-mediated gastroenterocolitis in an infant exposed to pembrolizumab in utero [103] and congenital hypothyroidism in a preterm infant exposed to nivolumab at conception [104]. In contrast, conception following an adequate washout interval (2–24 months) after IO treatment resulted in healthy pregnancies without immune complications [82].

#### Clinical Take-Home Message

The effects of IO on fertility remain poorly defined. Key questions include whether prior IO exposure increases risks of gestational complications or alters foetal immune development (Table 5) [105].

**Table 5:** Summary of IO therapy on ovarian function, fertility and pregnancy

	Immune Checkpoint Inhibitors (e.g. pembrolizumab, nivolumab, ipilimumab, atezolizumab)
Mechanism of action	Monoclonal antibodies targeting immune checkpoints (PD-1, PD-L1, CTLA-4) to restore T-cell–mediated antitumour activity and enhance immune response against cancer cells
Endocrine effect	Can cause immune-mediated endocrinopathies affecting hypothalamic–pituitary–gonadal, thyroid, and adrenal axes. Reported events include thyroiditis (9%), hypophysitis (1–2%), and adrenal insufficiency (< 2%)
Ovarian function effect	Preclinical data suggest immune-mediated primordial follicle loss with PD-1/CTLA-4 blockade. Limited clinical data show no additional AMH or FSH suppression when pembrolizumab is added to chemotherapy. Possible long-term immune-related ovarian dysfunction remains unconfirmed
Fertility and ovarian reserve	Sparse clinical evidence. No consistent human data demonstrating direct ovarian toxicity. Indirect impairment possible through hypophysitis or other endocrine toxicities
Pregnancy and neonatal outcomes	Pregnancy during IO exposure associated with immune-mediated foetal and maternal complications (miscarriage, oligohydramnios, neonatal immune dysfunction). Case reports describe neonatal enterocolitis and hypothyroidism. Conception after appropriate washout (≥ 12 months recommended) appears safe. Pregnancy should be avoided during and for ≥ 5 months post-therapy per NCCN/ASCO/ESMO guidelines

This table provides a narrative summary of key reproductive findings across studies. Detailed study characteristics, methodologies, and original data sources are cited and discussed in the corresponding sections of the main text

## PARP Inhibitors

PARP enzymes—primarily PARP-1 and PARP-2—are nuclear proteins that recognize single-strand DNA breaks and initiate base excision repair [106]. Inhibition of PARP results in accumulation of double-strand breaks that are lethal in homologous recombination (HR)–deficient cells, such as those harbouring BRCA1 or BRCA2 mutations, a process known as synthetic lethality [107]. Germline BRCA1/2 mutations occur in ~ 5% of unselected BC and confer a high lifetime risk of disease (~ 70–80%), with enrichment in triple-negative breast cancer (TNBC) and younger patient populations, making fertility preservation a key consideration [108–110].

PARP inhibitors (PARPi) such as olaparib and talazoparib exploit synthetic lethality by inhibiting PARP catalytic activity and trapping PARP–DNA complexes, leading to replication stress and cytotoxicity [111]. These agents are now standard of care in germline BRCA-mutated breast cancer in both the adjuvant and metastatic settings. In the OlympiA trial, one year of adjuvant olaparib improved invasive disease-free survival by 7.3% and overall survival by 3.4% at four years [112].

### PARP Inhibitors and Ovarian Function – Preclinical Data

Current knowledge on PARP inhibitors and gonadotoxicity is derived from animal studies. PARP-1 is expressed in oocytes and plays a role in maintaining genomic stability during folliculogenesis [113, 114]. In mice exposed to olaparib post-chemotherapy, primordial follicle counts fell by 36% compared with controls, although AMH and oestrogen levels were unchanged [115]. In vitro studies further demonstrate impaired granulosa cell function, including altered morphology and reduced oestradiol production [116]. PARP inhibition also reduced oocyte maturation and in vitro fertilisation rates in animal models [116, 117]. Importantly, BRCA1—but not BRCA2—deficiency itself is associated with reduced primordial follicle numbers and increased susceptibility to DNA damage [118]. A BRCA1-mutant rat model showed follicle loss, subfertility, and oxidative stress following olaparib, with increased susceptibility to chemotherapy [119].

### PARP Inhibitors—Embryonic Development and Pregnancy, Preclinical Data

PARP enzymes are critical for embryonic development. PARP1 is upregulated during implantation, while PARP2 contributes to blastocyst receptivity; inhibition of these pathways impairs implantation and early embryogenesis [120, 121]. Embryos exposed to PARP inhibitors exhibit developmental arrest, and PARP1/2 double-knockout mice are non-viable, arresting at the preimplantation stage [122, 123].

### BRCA Mutations and Baseline Fertility Considerations

Fertility concerns extend to BRCA carriers independent of PARPi use. Meta-analyses have demonstrated a 35% reduction in ovarian reserve in BRCA1—but not BRCA2—carriers [124, 125]. Consistently, BRCA1 carriers exhibit lower oocyte yield during stimulation and fewer primordial follicles with increased DNA damage [126–128]. They may also experience earlier menopause, with chemotherapy further accelerating ovarian decline [129–131]. The combined impact of germline *BRCA* mutations and systemic therapies, including PARP inhibitors, on long-term fertility outcomes remains incompletely defined [132]. Importantly, a large international retrospective cohort study involving more than 1400 BC survivors with germline *BRCA1/2* pathogenic variants demonstrated the safety of assisted reproductive techniques when natural conception is not achievable, supporting their use in this population [133].

### PARP Inhibitors – Clinical Considerations and Safety

While successful conception during PARPi therapy has been reported [134], preclinical data raise caution regarding reproductive safety (Table 6). All PARPi are contraindicated in pregnancy, with recommended washout intervals of six months for olaparib and seven months for talazoparib [113].

**Table 6:** Summary of PARP inhibitor therapy on ovarian function, fertility and pregnancy

	PARP inhibitors (e.g. talazoparib, olaparib)
Mechanism of action	Inhibit PARP-1/2, key enzymes in base-excision repair. Block catalytic activity and trap PARP–DNA complexes, causing replication stress and lethal double-strand breaks in homologous-recombination–deficient cells (e.g., BRCA1/2 mutations)
Endocrine effect	No confirmed clinical endocrine toxicity. Preclinical studies show altered granulosa-cell steroidogenesis and impaired oestradiol output, but human endocrine disruption has not been demonstrated
Ovarian function effect	PARP enzymes support oocyte genomic stability. Animal models show reduced primordial follicles after olaparib, impaired granulosa-cell function, and compromised oocyte maturation and fertilisation. BRCA1-mutant models display heightened follicle loss and susceptibility to DNA damage
Fertility and ovarian reserve	Human data on PARPi gonadotoxicity are lacking. BRCA1 carriers already have reduced ovarian reserve and earlier menopause, complicating attribution of direct drug effects. Preclinical evidence suggests possible fertility impairment, but real-world reproductive impact is unknown. Rare pregnancies during therapy have been documented
Pregnancy and neonatal outcomes	PARP enzymes are essential for embryogenesis and implantation. Embryos exposed to PARPi show developmental arrest; PARP1/2 loss is embryonically lethal. All PARPi are contraindicated in pregnancy. Recommended washout: **6 months (olaparib)**, **7 months (talazoparib)**

This table provides a narrative summary of key reproductive findings across studies. Detailed study characteristics, methodologies, and original data sources are cited and discussed in the corresponding sections of the main text

#### Clinical Take-Home Message

PARP inhibitors may adversely affect ovarian reserve and embryonic development based on preclinical evidence, although clinical data remain lacking; therefore, a precautionary approach with early fertility counselling and avoidance of pregnancy during treatment is recommended.

## CDK4/6 Inhibitors

CDK4/6 inhibitors—including ribociclib, palbociclib, and abemaciclib—have transformed treatment for HR-positive BC in both adjuvant and metastatic settings [135, 136]. In early BC, abemaciclib is approved for high-risk, node-positive disease [137]. The monarchE trial demonstrated a 6.4% absolute improvement in disease-free survival with abemaciclib plus endocrine therapy [138], increasing to 7.4% among premenopausal women [139]. Ribociclib was also recently approved in the adjuvant setting based on NATALEE trial with an absolute improvement in disease-free survival of 3.3% at 3 years [140]. Mechanistically, CDK4/6 inhibition prevents retinoblastoma protein phosphorylation, halting G1–S transition and inducing senescence [141].

### CDK4/6 Inhibitors and Gonadotoxicity – Preclinical Data

Preclinical evidence indicates a role of CDK4/6 in ovarian function [142–144]. Both CDK4 and CDK6 are expressed in murine oocytes [142]. CDK4-deficient mice are sterile, exhibiting small ovaries, reduced ovarian efficiency, low progesterone, and implantation failure [143, 144]. Combined CDK4/6 knockout in mice results in embryonic lethality [145]. In toxicology animal models, abemaciclib reduced progesterone and testosterone but slightly increased oestradiol levels [146]. In vitro studies demonstrate that exposure to CDK4/6 inhibitors induces replication stress and apoptosis in both cancerous and non-cancerous cells [147], though it remains unclear whether these effects extend to human ovarian tissue. Interestingly, palbociclib may exert a protective effect against oxidative stress-induced apoptosis in granulosa cells [148].

### CDK4/6 Inhibitors and Gonadotoxicity – Clinical Data

Clinical data are limited. The PENELOPE-B trial, which evaluated palbociclib in high-risk early BC, found no significant changes in FSH, oestradiol, or AMH levels following treatment, although AMH remained low after prior chemotherapy [149].

### CDK4/6 Inhibitors and Pregnancy – Preclinical Data

With regard to conception and pregnancy, exposure to palbociclib and ribociclib was associated with reduced maternal and foetal weights [150]. Ribociclib crosses the placental barrier and is secreted into milk in animal models [151]. FDA preclinical data also highlight potential foetal toxicity with all CDK4/6 inhibitors, including skeletal malformations, anaemia, and intrauterine death, warranting contraception during and after therapy [152].

#### Clinical Take-Home Message

Research on the gonadotoxicity of CKD4/6 inhibitors is lacking and mostly limited to animal studies. However, available data suggest a potential impact on ovarian tissue, fertility and pregnancy (Table 7).

**Table 7:** Summary of CDK4/6 inhibitor therapy on ovarian function, fertility and pregnancy

	CDK4/6 inhibitor (e.g. abemaciclib, palbociclib, ribociclib)
Mechanism of action	Block CDK4/6-mediated phosphorylation of retinoblastoma protein, arresting the G1–S transition and inducing senescence. Integral in HR-positive early and metastatic BC treatment
Endocrine effect	No established endocrine-axis toxicity in humans. Animal studies show reduced progesterone/testosterone and mild increases in oestradiol with abemaciclib, but clinical relevance remains uncertain
Ovarian function effect	CDK4/6 proteins are expressed in oocytes. CDK4-deficient mice are infertile, showing impaired ovulation, low progesterone, and implantation failure. In vitro, CDK4/6 inhibition can trigger replication stress; however, palbociclib may protect granulosa cells from oxidative injury. Human ovarian effects remain largely undefined
Fertility and ovarian reserve	Limited clinical evidence. In PENELOPE-B, palbociclib did not significantly alter AMH, FSH, or oestradiol (though most patients had prior chemotherapy). Overall fertility impact in humans is unknown
Pregnancy and neonatal outcomes	Preclinical data indicate class-wide embryo–foetal toxicity, including reduced foetal growth, skeletal defects, anaemia, and foetal loss. Ribociclib crosses the placenta and enters milk. Effective contraception is required during therapy and for an appropriate post-treatment interval

This table provides a narrative summary of key reproductive findings across studies. Detailed study characteristics, methodologies, and original data sources are cited and discussed in the corresponding sections of the main text

## Conclusions

The rapid integration of novel systemic therapies—including HER2-targeted monoclonal antibodies and antibody–drug conjugates, immunotherapy, CDK4/6 inhibitors, and PARP inhibitors—has dramatically improved survival in young women with BC. However, their impact on fertility and pregnancy remains incompletely defined, with significant variability in gonadotoxic potential across drug classes.

Current evidence highlights divergent risks across drug classes. Endocrine therapies primarily exert transient effects on ovarian reserve, with tamoxifen posing specific risks related to foetal exposure rather than ovarian toxicity. HER2-targeted monoclonal antibodies are relatively less gonadotoxic than chemotherapy, with some data suggesting a protective effect on ovarian reserve; however, they are contraindicated in pregnancy. The antibody–drug conjugate T-DM1 may also be less damaging compared with standard chemotherapy regimens. Immunotherapy introduces unique concerns, including immune-mediated ovarian injury and delayed-onset endocrinopathies, raising questions about optimal timing of conception. PARP inhibitors and CDK4/6 inhibitors are widely used in young patients, yet their effects on fertility remain almost entirely undefined beyond preclinical and regulatory data, underscoring a major translational gap.

Despite preclinical evidence, no human studies have directly evaluated the impact of PARP inhibitors, other antibody–drug conjugates such as T-DXd and CDK4/6 inhibitors on ovarian reserve or fertility outcomes. Therefore, conclusions regarding the reproductive toxicity of these agents are necessarily informed by preclinical and translational models rather than direct clinical evidence. While these studies provide important mechanistic insights and signal potential risks to ovarian physiology, their clinical relevance remains uncertain in the absence of prospective human data. Accordingly, interpretation of animal and in vitro findings should be approached with caution, and direct extrapolation to fertility or pregnancy outcomes in women should be avoided. Where available, clinical observations are highlighted separately and prioritized in this review, underscoring the need for carefully designed prospective studies to define the true reproductive impact of these therapies.

Artificial intelligence (AI)–driven predictive models represent a promising avenue to refine individualized oncofertility counselling. Traditional risk stratification for treatment-related amenorrhea and infertility relies on clinical variables such as age, chemotherapy regimen, and baseline ovarian reserve; however, these approaches lack precision at the individual level. Recent advances in machine learning have enabled the development of predictive models integrating multidimensional clinical and biological data to estimate the risk of treatment-related amenorrhea with improved accuracy [153]. Such tools may facilitate personalized decision-making, including the selection of fertility preservation strategies and optimization of treatment sequencing. Importantly, future models should incorporate treatment-specific variables related to modern systemic therapies—including targeted agents, antibody–drug conjugates, and immunotherapy—as well as longitudinal reproductive outcomes. This aligns with broader efforts to integrate survivorship-focused endpoints and innovative analytical approaches into breast cancer research [5]. Prospective validation and integration into clinical workflows will be essential to translate these approaches into routine practice.

Looking ahead, the integration of standardized reproductive endpoints into oncology trials, development of preclinical models that better capture human ovarian physiology, and establishment of prospective fertility registries are essential. Only by systematically evaluating both short- and long-term reproductive consequences can oncologists provide evidence-based counselling, balance oncologic efficacy with quality-of-life priorities, and ensure that survivorship includes the possibility of parenthood for those who desire it.

## Key References

- Mannion, S. et al. Prevalence and Impact of Fertility Concerns in Young Women with Breast Cancer. *Sci. Reports 2024 141* 2024, *14*, 4418.
  - This reference is of high importance as it demonstrates the prevalence of fertility concerns and their significant influence on treatment decision-making in a contemporary cohort of young women with breast cancer.
- Soldato, D. et al. The Future of Breast Cancer Research in the Survivorship Field. *Oncol. Ther.* 2023, *11*, 199
  - This reference is of high importance as it highlights the long-term impact of breast cancer treatments—including effects on fertility—on survivorship in young patients.
- Kim, A.E. et al. Minding the Bathwater: Fertility and Reproductive Toxicity in the Age of Immuno-Oncology. *JCO Oncol. Pract.* 2022, *18*, 815.
  - This reference is of importance as it provides a detailed analysis of gonadotoxicity and fetomaternal risks associated with immune checkpoint inhibitors, an increasingly relevant issue given the expanding use of immunotherapy in breast cancer patients.

## Data Availability

No datasets were generated or analysed during the current study.
